# Drug repositioning prediction for psoriasis using the adverse event reporting database

**DOI:** 10.3389/fmed.2023.1159453

**Published:** 2023-03-23

**Authors:** Minoh Ko, Jung Mi Oh, In-Wha Kim

**Affiliations:** ^1^College of Pharmacy, Seoul National University, Seoul, Republic of Korea; ^2^Research Institute of Pharmaceutical Sciences, Seoul National University, Seoul, Republic of Korea

**Keywords:** drug repositioning, adverse events, disproportionality, signals, psoriasis

## Abstract

**Introduction:**

Inverse signals produced from disproportional analyses using spontaneous drug adverse event reports can be used for drug repositioning purposes. The purpose of this study is to predict drug candidates using a computational method that integrates reported drug adverse event data, disease-specific gene expression profiles, and drug-induced gene expression profiles.

**Methods:**

Drug and adverse events from 2015 through 2020 were downloaded from the United States Food and Drug Administration Adverse Event Reporting System (FAERS). The reporting odds ratio (ROR), information component (IC) and empirical Bayes geometric mean (EBGM) were used to calculate the inverse signals. Psoriasis was selected as the target disease. Disease specific gene expression profiles were obtained by the meta-analysis of the Gene Expression Omnibus (GEO). The reverse gene expression scores were calculated using the Library of Integrated Network-based Cellular Signatures (LINCS) and their correlations with the inverse signals were obtained.

**Results:**

Reversal genes and the candidate compounds were identified. Additionally, these correlations were validated using the relationship between the reverse gene expression scores and the half-maximal inhibitory concentration (IC50) values from the Chemical European Molecular Biology Laboratory (ChEMBL).

**Conclusion:**

Inverse signals produced from a disproportional analysis can be used for drug repositioning and to predict drug candidates against psoriasis.

## 1. Introduction

Drug repositioning or repurposing, which involves exploring new therapeutic applications for approved drugs, has become an increasingly effective strategy for drug development (1). The advantage of drug repositioning approaches is that the development risk can decrease, with the drug rapidly entering late-stage clinical trials, thereby reducing the developing cost and time required to develop a novel drug (2, 3). The gene expression signature-based method is a more recent approach that classifies drugs or diseases based on their gene expression signatures as compared to traditional methods (4, 5). Additionally, computational drug repositioning methods that operate on the signature reversion principle based on disease-specific or drug-induced gene expression were developed and established by integrated omics technologies (6, 7).

Adverse drug events, i.e., unwanted harmful reactions resulting from the use of medicines and the identification of unintended drug effects, can create the opportunity for drug repositioning. Post-market surveillance of approved drugs is essential for providing assurances regarding the efficacy as well as safety of medicines. Spontaneous drug adverse event reporting (AER) has wide coverage, meaning that rare or serious adverse reactions not detected during clinical trials before approval may be revealed in patient populations during the post-marketing phase (8). AER is considered to be the mainstay of adverse drug reaction reporting systems and is a valuable source of real-world data about post-market drug safety. Additionally, the data from these systems can be used to evaluate potential risks associated with drugs in pharmacovigilance studies using various analytical methods. Currently, the use of inverse signals generated from a disproportional analysis of spontaneous drug AER is being suggested as a promising approach for drug (9, 10).

Psoriasis is a chronic, multifactorial, refractory, and inflammatory disease. It mainly affects the skin but can also affect other parts of the body. The pathophysiology of psoriasis is complicated and not fully understood. It is known that psoriasis is associated with immunological and genetic susceptibility and environmental triggers such as infection, stress, smoking, obesity, and alcohol consumption (11). Currently, immunotherapies are the main treatment methods for psoriasis (12). However, many patients with psoriasis still face challenges in managing the disease due to its chronic and recurrent nature, as well as potential side effects such as increased risk of severe infections, immunologically mediated allergic reactions, and other unwanted responses (13). This highlights the need for continued research and the exploration of new and more effective treatment options for psoriasis. Therefore, it is necessary to continue to explore or develop new effective treatments for psoriasis. From this point of view, drug repositioning is an efficient and promising drug discovery strategy to provide benefits to most patients with psoriasis.

Consequently, the purpose of this study is to propose a new systematic approach by which to identify potential candidate drugs as a treatment for psoriasis through a computational drug repositioning method that combines drug adverse event report data with disease-specific and drug-induced gene expression profiles.

## 2. Methods and materials

### 2.1. Data collection and preprocessing of adverse events

**Figure 1:**
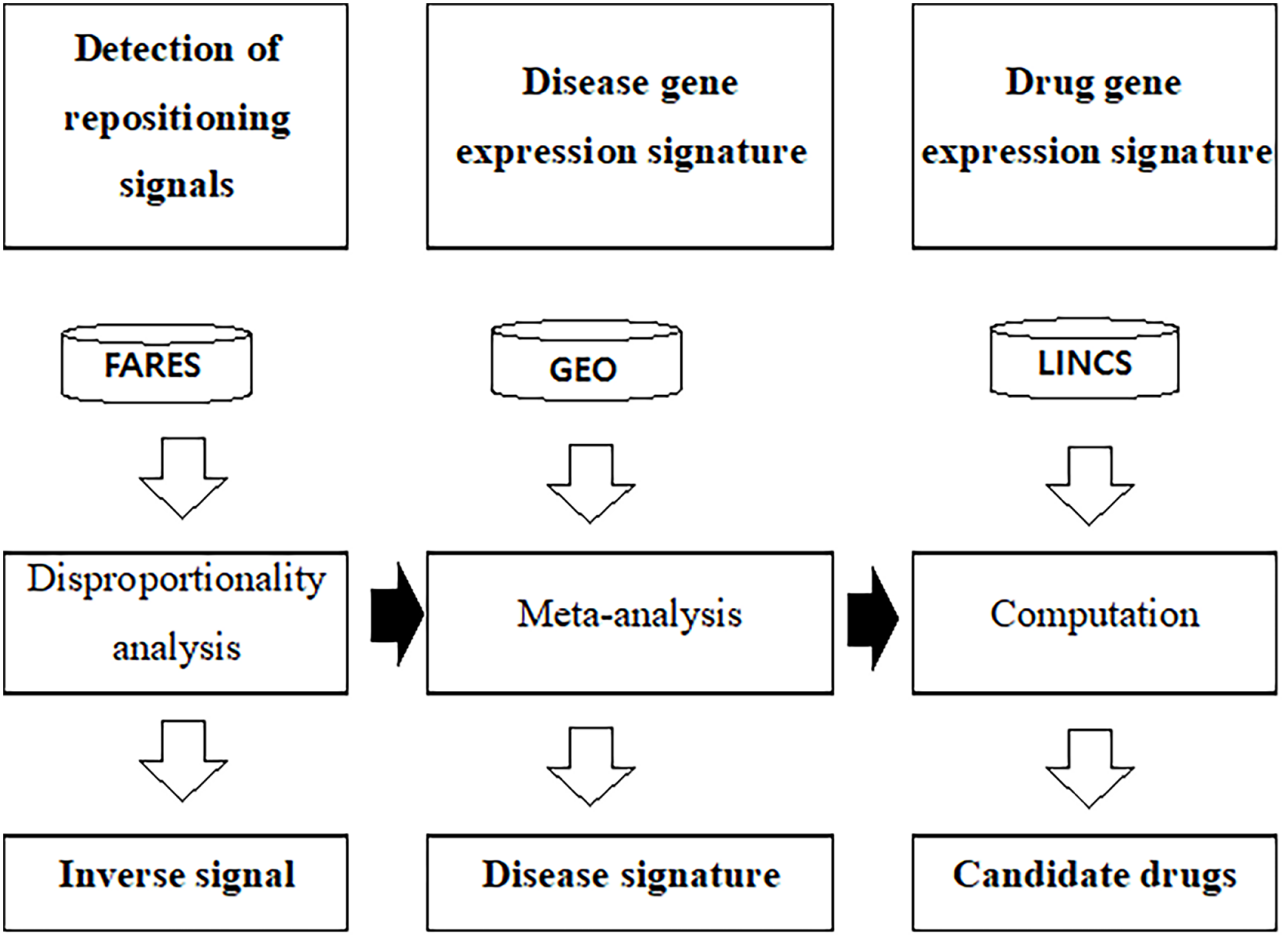
Workflow to predict drug candidates using gene expression profiles and adverse event data. FAERS was used to calculate the inverse signal. The public GEO database used to calculate disease specific gene expression signature. LINCS L1000 was used as the drug signature database. ChEMBL was used as the drug efficacy database. FAERS, U.S. Food and Drug Administration adverse event reporting systems; GEO, Gene Expression Omnibus; LINCS, The Library of Integrated Network-Based Cellular Signature.

The workflow for the screening of compounds using the inverse signal, disease signature, and drug signature is presented in Figure 1. Adverse event cases reported from 2015 to 2020 were downloaded from the U.S. Food and Drug Administration (FDA) adverse event reporting systems (FAERS) database. Duplicated case reports overlapping in fields, including the case number and event date, were removed and the report of the most recent case number remained according to the FDA’s recommendations (14). Adverse events in the FAERS database are coded with the preferred term (PT) of the Medical Dictionary for Regulatory Activities (MedDRA) (15). Medicines identified as primary suspect drugs were selected for further analyses. Adverse events that were classified as “drug ineffective” or “medication error” were excluded. Additionally, combination drugs that contained two or more active ingredients in a single dosage form were excluded. FAERS data are freely downloadable and do not contain patients’ personal information. Therefore, ethics approval and informed consent were not required for this study.

### 2.2. Disproportionality analysis and calculations of inverse signals

MedDRA PT was initially used for the disproportionality analysis in order to calculate the signal values. The reporting odds ratio (ROR) (16), the information component (IC) (17), and empirical Bayes geometric mean (EBGM) algorithms (18) were applied to calculate the disproportionality outcomes. The ROR values were calculated as the ratio of the odds of the reporting of one specific adverse event versus all other events for a given drug, compared to the reporting odds for all other drugs (16). The IC values can be calculated by the logarithm of the ratio of the observed rate of a specific drug-adverse event reporting pair, to its expected rate (17). EBGM values are adjusted estimates of observed/expected relative reporting ratios after Bayesian shrinkage corrections (19). An inverse association for the ROR was defined, when the upper limit of the 95% confidence interval (CI) (RORUCI) was <1 (9). An inverse association for the IC was defined if the upper limit of the 95% CI (IC975) was <0 (9). For the EBGM, an inverse association was defined if the upper limit of the 90% CI (EBGM95) was <1. All data mining and analyses were conducted using SAS version 9.4 (SAS Institute Inc., Cary, NC, United States). MedDRA PT was mapped to the Tenth Revision of the International Classification of Diseases and Related Health Problems (ICD-10) codes with BioPortal, a repository of biomedical ontologies (20). The terms corresponding to symptoms, signs, and infective diseases were excluded. Psoriasis was selected as the target disease because it is the refractory diagnostic disease associated with the most reported drugs. Adverse events corresponding to psoriasis included pustular psoriasis, dermatitis psoriasiform, erythrodermic psoriasis, nail psoriasis, Guttate psoriasis, rebounded psoriasis, and paradoxical psoriasis, psoriasis area severity index increased. The inverse signal, calculated RORUCI, IC975 value, or the EBGM95 value was used to represent the drug activity.

### 2.3. Data collection and preprocessing of disease gene expression data

A search was conducted for expression data related to psoriasis in the Gene Expression Omnibus (GEO) database hosted by the National Center for Biotechnology Information (NCBI) using text keyword ‘psoriasis’ in August of 2021. Next, the datasets were filtered with ‘Homo sapiens’ in the organism field and ‘expression profiling by array’ or ‘high throughput sequencing’ in the study type field. Information pertaining to the accession number, platforms, organisms, experiment type, and the number of samples with psoriatic or normal lesions, were collected from each of the identified datasets. After excluding psoriatic arthritis from datasets, the original gene expression datasets of psoriatic and normal lesions from psoriatic patients were downloaded. The logarithmic transformations of expression levels were normalized. If multiple probes were mapped to the same gene, the probe with the highest interquartile range was chosen. The quality of the gene expression data was assessed with the R package MetaQC (21). MetaQC calculated quantitative quality control (QC) measures, in this case, the internal QC, external QC, accuracy QC, and consistency QC. The standardized mean rank summary score for each dataset was calculated based on these QC measures.

### 2.4. Identification of differently expressed genes

DEGs associated with psoriasis were identified by the meta-analysis R package MetaDE (22). The *p* values for each dataset were calculated *via* a moderated *t*-statistic test. A fixed effect model was used to combine *p* values and effect sizes for the meta-analysis (23). The DEGs were used as disease signatures for a further analysis.

### 2.5. Data collection of compound-induced gene expression data

Here, the 345,976 drug signatures and 12,328 genes including 978 landmark genes for a variety of compounds, and L1000 assay data were downloaded from the Library of Integrated Network-based Cellular Signatures (LINCS) data portal in August of 2021 (24). Information pertaining to the cell lines, drugs, dosages, and time points was collected.

### 2.6. Computation of reverse gene expression scores

This study uses the method of Chen et al. and the description of the methods partly reproduces their wording (6). Briefly, an enrichment score of a gene expression level associated with disease was computed based on their rank of the DEGs. Genes were ranked based on their expression levels in each drug signature. Reverse score values represent a reversal correlation between DEGs associated with compounds and a disease. Therefore, a lower negative value of the reverse score is an indication of a higher likelihood to change to reverse the gene expression associated with the disease, and vice versa. This resulted in more than one reverse scores for each compound that could reverse the expression levels associated with the disease. Given these variations, summarized reverse scores of gene expression were weighted and calculated. Multiple drug gene expression associated with each compound may depend on the experimental conditions, including the cell lines, dosages, and time points of the drugs. The condition with a drug concentration of 10 μM and treatment time of 24 h was set as the reference condition (6).

### 2.7. Identification of candidate compounds and reversed genes

Each gene was ranked by its expression value. Upregulated genes were ranked toward the top, whereas downregulated genes were ranked toward the bottom. The genes showing reversal in their expression were determined by the leave-one-compound-out cross-validation process (25). Each compound was systemically excluded once in turn and only once from the dataset, and reversed genes were then identified using the approach described above. In all trials, genes with false discovery rate-adjusted *p* values of less than 0.25 were considered as reversal genes.

### 2.8. Validation using the half-maximal inhibitory concentration (IC50)

The IC50 levels of compounds for human were searched and downloaded from the Chemical European Molecular Biology Laboratory (ChEMBL) (26). Compounds in ChEMBL were manually mapped with those in LINCS based on InChIkeys. If multiple IC50 values were available for one compound, the corresponding median, minimum, or maximum value was calculated to search for the relationship between the reverse gene expression score and the IC50 value.

## 3. Results

### 3.1. Inverse signals

In total, 28,306,315 records were downloaded from FAERS for the time period of 2015 through 2020. After removing duplicate and ineffective medication records, 7,508,403 subjects remained (Supplementary Table S1). The proportion of subjects over the age of 64 was 21.78%, compared to the proportion of subjects under the age of 17 at 3.06%. There were a total of 21,659,660 drug-adverse event pairs, representing 4,957 drugs and 19,683 adverse events. A significant inverse association was detected in 175,097 (ROR UCI < 1), 403,809 (IC975 < 0), and 224,222 (EBGM95 < 1) combinations of drugs and adverse events. Of those PT adverse events, only 1,909 (9.70%) were mapped to ICD-10 codes using BioPortal. After excluding symptoms, signs, and infective diseases, psoriasis was selected as the target disease for drug repositioning because psoriasis as an adverse event was associated with the highest number of drugs. Drugs inversely associated with psoriasis are listed in Supplementary Table S2.

### 3.2. Disease gene expression signature

**Figure 2:**
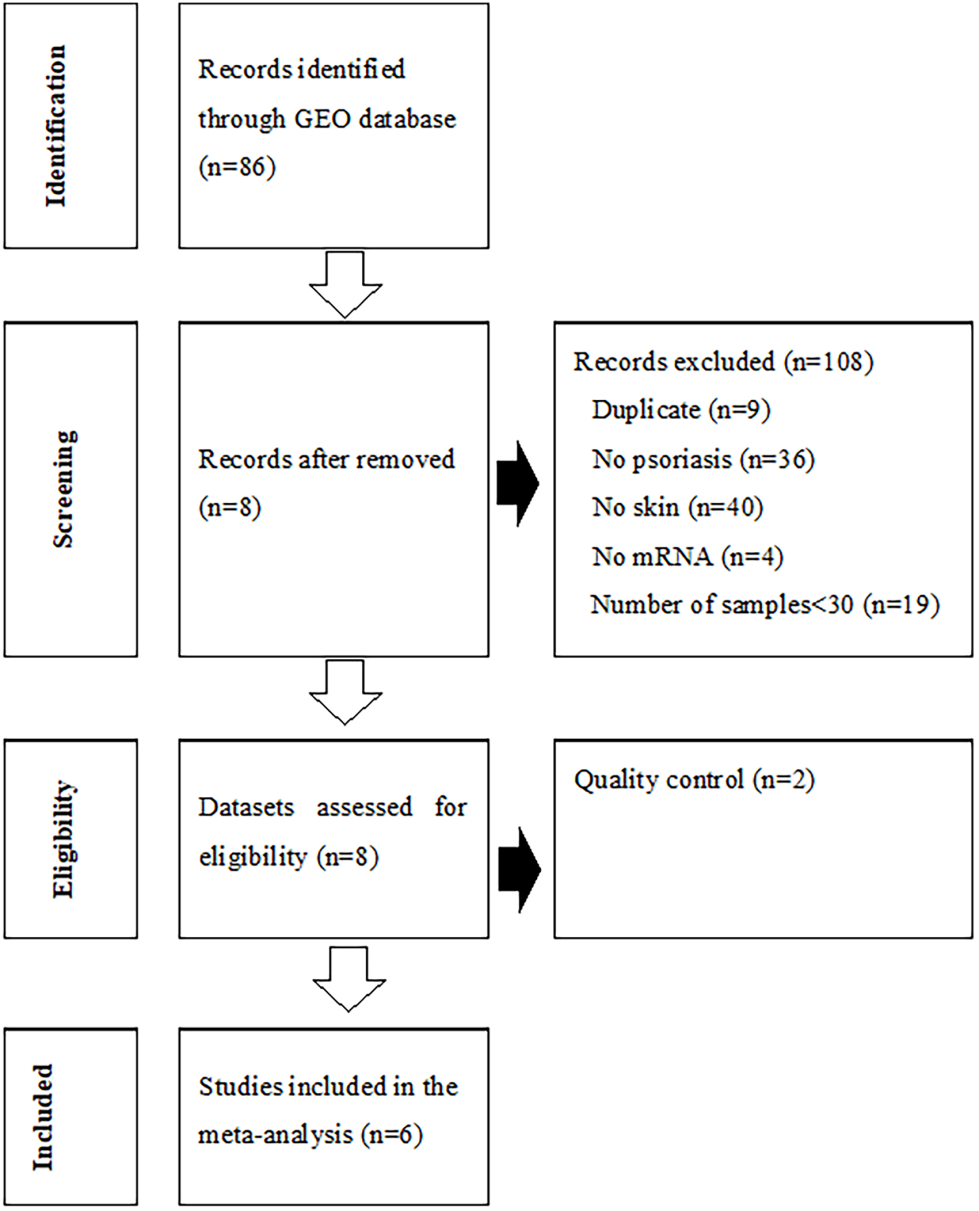
Flowchart of the process used to select gene expression datasets for the meta-analysis of psoriasis. GEO, Gene Expression Omnibus.

The process of dataset selection for psoriasis is shown in Figure 2. Our search of the GEO database yielded 116 GEO Series Experiments (GSEs). A number of datasets were excluded due to duplicate data (*n* = 9), a different disease (*n* = 36), a lack of skin samples (*n* = 40), a lack of mRNA expression data (*n* = 4), and studies with fewer than 30 samples (*n* = 19). The eight datasets of GSE78097, GSE79704, GSE85034, GSE109248, GSE117239, GSE117468, GSE121212, and GSE136757 were selected for the MetaQC analysis. The Information pertaining to of these datasets is summarized in Supplementary Table S3. The QC results of QC indicated that the GSE79704 and GSE109248 datasets had relatively low quality levels, and markedly deviating considerably ed. from the other six datasets (Supplementary Table S4, Supplementary Figure S1). Finally, selected six selected datasets (GSE78097, GSE85034, GSE117239, GSE117468, GSE121212, and GSE136757) containing 364 psoriatic lesions and 349 normal lesion were included in the disease signature analysis. Among the disease signatures, 206 genes showed increased expression levels in psoriatic lesions compared to control lesions (adjusted *p* < 0.001, log 2 (fold change) > 1.5), whereas 46 genes showed decreased expression levels in psoriatic lesion (adjusted *p* < 0.001, log 2 (fold change) < −2.0, Supplementary Figure S2). Seventy-four DEGs filtered *via* log 2 (fold change) > 2.5 or < −2.5 and adjusted *p* < 0.001 are listed in Supplementary Table S5.

### 3.3. Drug gene expression signature

**Figure 3:**
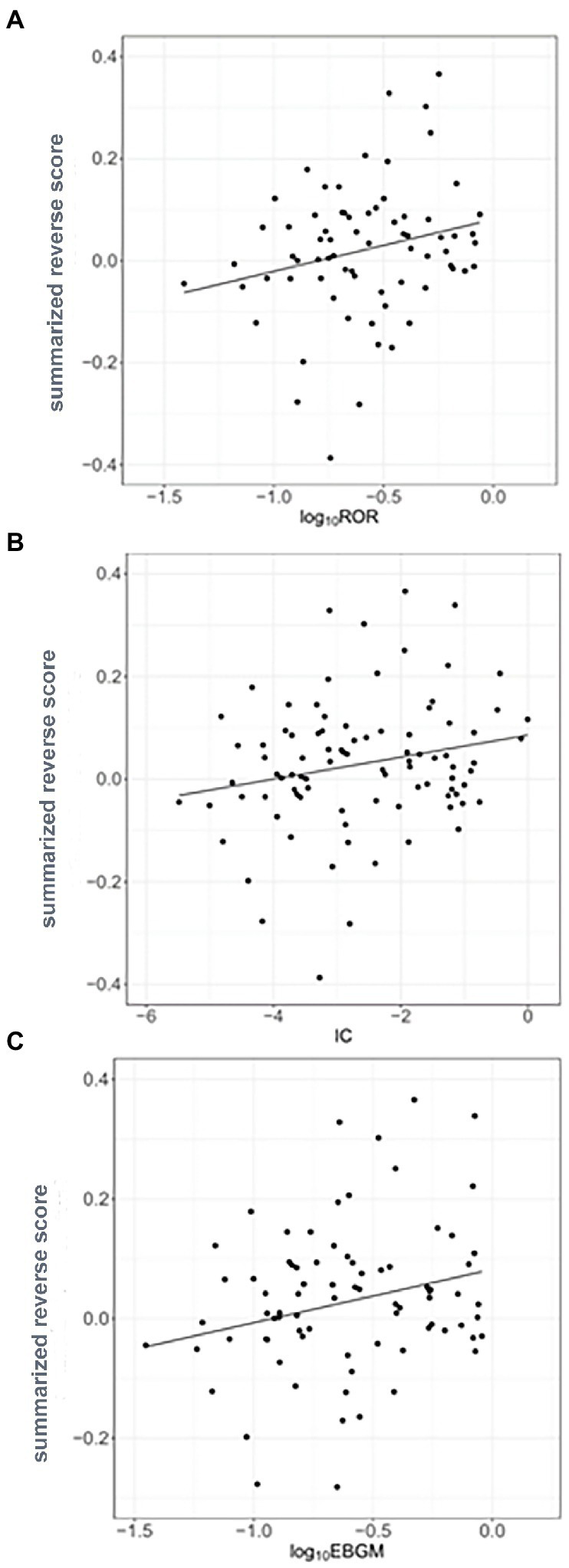
Correlation between the inverse signals and summarized reverse gene expression scores in psoriasis **(A)** Correlation between inverse signals from reporting odds ratio (ROR) and summarized reverse scores (Spearman’s correlation rho = 0.357, *p <* 0.05), **(B)** Correlation between inverse signals from information component (IC) and summarized reverse scores (Spearman’s correlation rho = 0.336, *p <* 0.05), and **(C)** Correlation between inverse signals from empirical Bayes geometric mean (EBGM) and summarized reverse scores (Spearman’s correlation rho = 0.315, *p <* 0.05). The line indicates linear regression line.

Reverse scores as drug signatures were computed by changes in the landmark gene expression levels of A375 human melanoma cell after 451 compound treatment from the LINCS data. Summarized reverse scores were computed by weighting the dosages and time points of the compounds and various cell lines. The calculated summarized reverse scores for each compound were significantly correlated with the inverse signals of ROR (Spearman correlation rho = 0.357 and *p* < 0.05; Figure 3A), IC (Spearman correlation rho = 0.336 and *p* < 0.05; Figure 3B), and EBGM (Spearman correlation rho = 0.315 and *p* < 0.05; Figure 3C).

### 3.4. Validation using the IC50s

**Figure 4:**
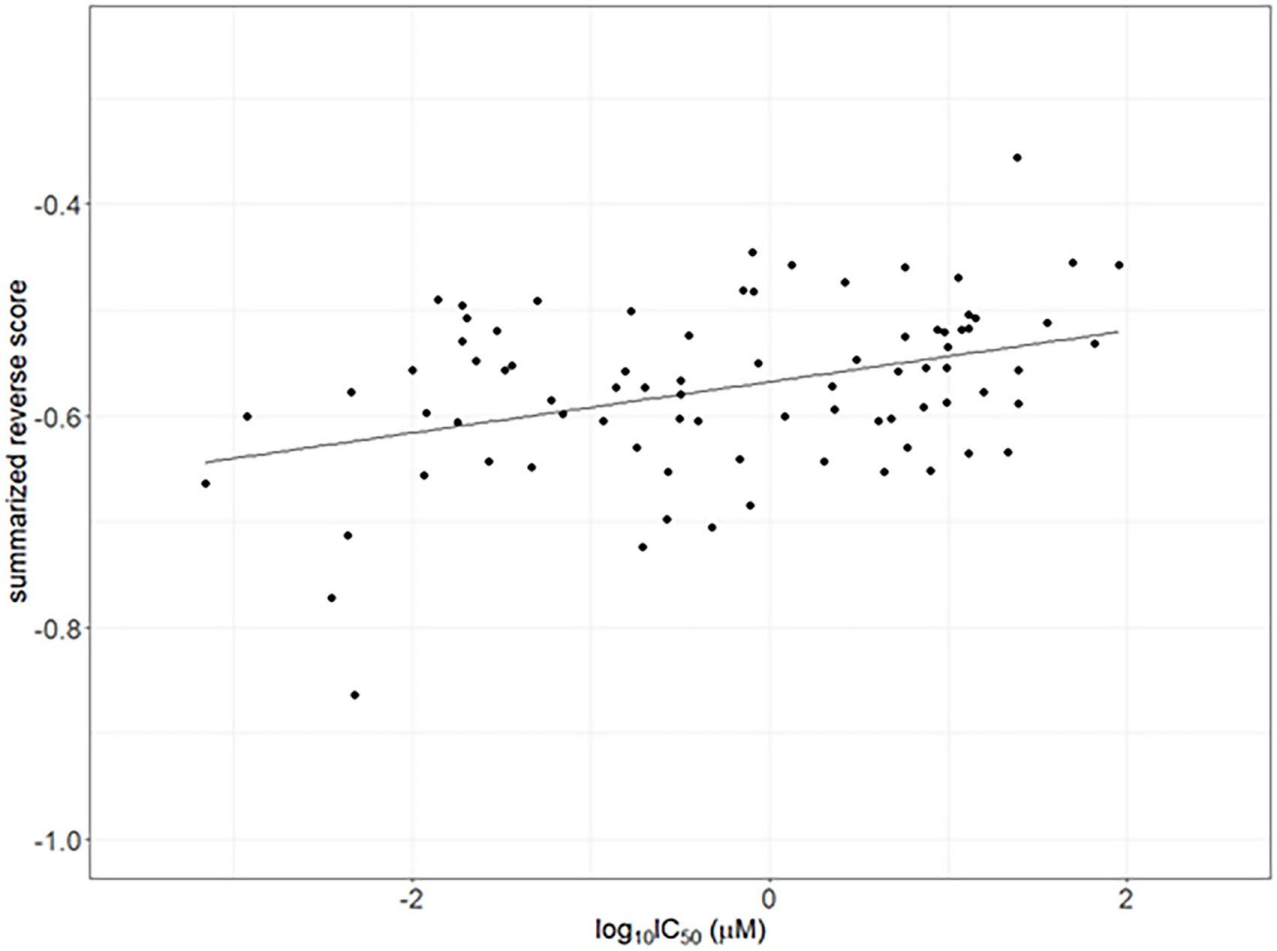
Correlation between the half-maximal inhibitory concentrations (IC50) as drug efficacy from ChemBL and summarized reverse gene expression scores in psoriasis. The line indicates linear regression line (Spearman correlation rho = 0.338 and *p* < 0.05).

The IC50 values for compounds having reversal scores in ChEMBL were used for validation. Calculated summarized reversal scores were significantly correlated with the median IC50 values for each compound (Spearman correlation rho = 0.338 and *p* < 0.05; Figure 4).

### 3.5. Reversed gene identification and compound predictions

**Figure 5:**
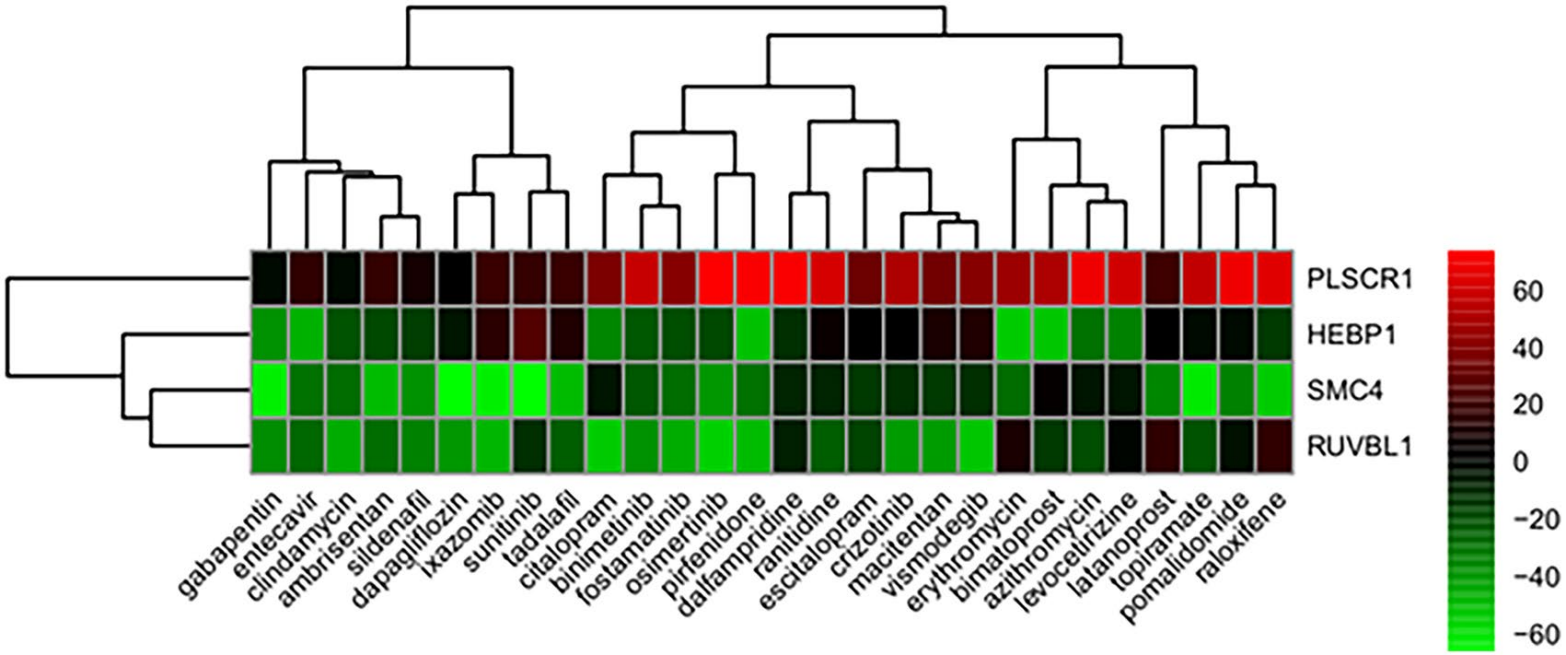
Genes showing reversed expression in response to treatments with compounds. Low and high rank suggest that the gene expression is down- and upregulated, respectively, by the corresponding compound. The heatmap indicates the relative position of a gene in ranked drug expression data. Position are normalized and compound columns are ordered according to inverse signal of ROR. Red and green colors indicate up- and down-regulation, respectively, after compound treatment.

From the correlation between the summarized reverse score values and drug activity outcomes, compounds having high potency levels for psoriasis were identified. Next, reversely expressed genes by compound were predicted by a leave-one-out cross-validation procedure. Only using the inverse signals of ROR, four genes showed significantly reversed expressions (Figure 5): (i) Phospholipid Scramblase 1 (*PLSCR1*), (ii) Structural Maintenance Of Chromosomes 4 (*SMC4*), (iii) Heme Binding Protein 1 (*HEBP1*), and (iv) RuvB Like AAA ATPase 1 (*RUVBL1*). The drugs against psoriasis identified here were the endothelin receptor antagonist, ambrisentan and macitentan; the prostaglandin analogs, bimatoprost and latanoprost; the immunosuppressants, pirfenidone and pomalidomide; the macrolide antibiotics, azithromycin and erythromycin; and the antineoplastic agents, binimetinib, crizotinib, ixazomib, osimertinib, sunitinib, and vismodegib.

## 4. Discussion

A number of methods have been proposed to predict new indications for drugs based on certain data sources. In this study, we used a computational method that integrates drug adverse event report data, disease-specific expression profiles and drug-induced expression profiles to predict drug candidates for psoriasis treatments.

FAERS used here is one of the largest repositories of spontaneously reported adverse events in the world and contains more than 21 million reports dating from 1968 to December 2020, with reports from multiple countries. FAERS is frequently used in relation to disproportionality analyses given its rapid updates and the fact that it contains data reported from multiple countries. In the present study, each inverse signal of ROR, IC, or EBGM calculated *via* disproportionality analyses was examined, as signals detected from adverse events have been shown to depend on certain algorithms (27). The IC method provided the highest number of inverse signals, while the ROR method provided the lowest. This is relevant because the ROR method provided the highest number of signals because it has the highest sensitivity compared to the IC and EBGM methods when using the FAERS database (27). The cut-off of the inverse signal for EBGM95 is not known, therefore, we attempted to determine an appropriate cut-off value. In fact, signal scores cannot be used to determine the rank order of drugs in terms of risk using AERS (27) due to underreporting. However, Hochberg et al. reported as a pilot study that differences in signal scores between similar drugs in the AERS database were in agreement with the differences in incidence rates of adverse events in several published studies (28).

The MedDRA PT level terms were grouped into Standardized MedDRA Queries (SMQ) due to the variability in the PTs chosen to describe the same adverse symptom (29). However, since the number of mapped of MedDRA PT terms with ICD-10 was higher than that of SMQs with ICD-10, MedDRA PT terms were used in the disproportionality analyses in our study. It is known that SMQ terms are related to a defined medical condition or area of interest and are intended to support case identification efforts, but do not cover all medical conditions that may be related to a drug or have the required specificity (29).

The calculated summarized reverse scores were significantly correlated with inverse signals obtained from RORUCI, IC975, and EBGM95 for each compound for psoriasis. This indicates that combining disease gene expression profiles and drug activities with inverse signals can be used to predict drug indications. To the best of the author’s knowledge, this study addressed drug repositioning for the first time using a computational approach with an inverse signal from an adverse event reporting system database to represent real-world data and reversal gene expression scores in relation to psoriasis.

In our study, four genes were found to have reversed expression levels and thus may be potential therapeutic targets for psoriasis. The expression levels of phospholipid scramblase 1 (*PLSCR1*), heme binding protein 1 (*HEBP1*), structural maintenance of chromosomes 4 (*SMC4*), and RuvB like AAA ATPase 1 (*RUVBL1*) genes were shown to be reversed by candidate compounds for psoriasis using the RORUCI values. *PLSCR1* was shown to play an important role in the interleukin (IL)-36/interferon-I axis contributing to psoriasis (30). The F2L peptide encoded by *HEBP1* promotes calcium mobilization and chemotaxis in monocytes and dendritic cells, which would contribute to tissue repair and control of the inflammatory process (31). *SMC4* is an essential gene that encodes a member of a ubiquitous family of chromosome-associated ATPases, and ubiquitination is viewed as a key process in psoriasis pathology (32). *RUVBL1* belongs to the AAA+ ATPase family and plays an essential role in the maintenance of genomic stability, cellular proliferation, and cell cycle progression (33).

Our study identified several repositionable candidate drugs previously known to be effective for psoriasis clinically or in preclinical studies. Macrolide antibiotics have been shown to affect the production and release of proinflammatory cytokines (34). Accordingly, topical forms of clindamycin have been used off-label for psoriasis. It was reported in relation to an antiepileptic, topiramate, that the psoriasis area and severity index score were decreased in a pilot study (35). A selective estrogen receptor modulator, raloxifene, was suggested to be potentially effective for the treatment of psoriasis as an inhibitor of IL-12p40 and tumor necrosis factor (TNF)-α (36). Ambrisentan, an endothelin receptor A antagonist, significantly attenuated the development of imiquimod-induced psoriasiform dermatitis in a mouse model (37). Tadalafil was the first phosphodiesterase (PDE) 5 inhibitor with FDA approval for benign prostatic hyperplasia, and selective PDE inhibitors have shown an anti-inflammation effect and promise during treatments of psoriasis and psoriatic arthritis (38). Pomalidomide was also predicted to be a potential anti-psoriasis drug because it inhibits TNF-α production (39). One case report found that gabapentin improved psoriasis (40), and a pilot study showed that topiramate decreased psoriasis areas and severity index levels (35). Preclinical and clinical evaluations for several kinase inhibitors are ongoing in relation to psoriasis (41, 42). A spleen tyrosine kinase inhibitor, R406, an active metabolite of fostamatinib, was shown to suppress psoriasis-like inflammation in mice (43).

The genetic mechanisms of psoriasis are complex, and various immune factors such as epidermal growth factors, nerve growth factors, chemokines, neuropeptides, adhesion factors, T-cell receptors, and abnormal activities of tyrosine kinases play critical roles in its pathogenesis. Therapeutic action and efficacy are most likely more complicated than simple relationships between gene expression patterns and drug activities. Therefore, our findings could be enhanced with preclinical investigations or clinical trials. Another limitation of our study is that the drugs studied in LINC data did not exist in the adverse drug reaction reporting system. Therefore, the identified drugs were limited to drugs that had been approved. However, conducting randomized clinical trials is not easy due to increases in the cost and trial duration when increasing the number of procedures. A computational approach with spontaneous adverse drug reaction reporting data may become a useful strategy by which to find new drug candidates for drug repositioning.

In summary, our computational method combined gene expression with inverse signals obtained from disproportional analyses of psoriasis to identify new drugs and target genes as psoriasis therapies. This method, as a repositioning approach, can also be used to predict the efficacy of new drug candidates to treat other diseases. This computational method can be broadly applied to other diseases for which reliable reports of adverse event data as real-world data are available.

## Data availability statement

The datasets generated and/or analyzed for this study can be found are available from the corresponding author on reasonable request.

## Author contributions

MK and I-WK analyzed the data and prepared the manuscript. I-WK and JO contributed to the conception and design of the study. All authors were engaged in commenting on the manuscript, read, and approved the final manuscript.

## Funding

This study was supported by the National Research Foundation of Korea grant funded by the Korea government (MSIT) (no. 2021R1A2C1006046).

## Conflict of interest

The authors declare that the research was conducted in the absence of any commercial or financial relationships that could be construed as a potential conflict of interest.

## Publisher’s note

All claims expressed in this article are solely those of the authors and do not necessarily represent those of their affiliated organizations, or those of the publisher, the editors and the reviewers. Any product that may be evaluated in this article, or claim that may be made by its manufacturer, is not guaranteed or endorsed by the publisher.
