# Supplementary material for: Drug repositioning prediction for psoriasis using the adverse event reporting database
Source: Front Med (Lausanne). 2023 Mar 23;10:1159453. doi: 10.3389/fmed.2023.1159453 (PMC10076533; doi:10.3389/fmed.2023.1159453)
Supplement: Supplementary file 1 [file Data_Sheet_1.DOCX]

Supplementary Material

Drug repositioning prediction for psoriasis using the adverse event reporting database

Minoh Ko, Jung Mi Oh, In-Wha Kim*

*** Correspondence:** In-Wha Kim: iwkim2@hanmail.net

# Supplementary Figures and Tables

.

## Supplementary Tables

Supplementary Table S1. Demographic characteristics of the study population from FAERS

| **Characteristics** | **Subjects (*N*=7,508,403)** |
| --- | --- |
| Age, *N* (%) |  |
| Less than 1 month | 13,317 (0.18) |
| 1 month to 2 years old | 25,705 (0.34) |
| 2 to 12 years old | 91,514 (1.22) |
| 12 to 17 years old | 98,894 (1.32) |
| 17 to 64 years old | 2,438,259 (32.47) |
| More than 64 years old | 1,635,393 (21.78) |
| Unknown, *N* (%) | 3,205,321 (42.69) |
| Gender, *N* (%) |  |
| Male | 2,568,522 (34.21) |
| Female | 4,006,972 (53.37) |
| Unknown | 932,879 (12.42) |
| Drug | 4,957 |
| Adverse event | 19,683 |

Supplementary Table S2. Signals of drugs associated with psoriasis from disproportional analyses using FAERS data*

| Drug | RORUCI | IC975 | EBGM95 | Drug | RORUCI | IC975 | EBGM95 |
| --- | --- | --- | --- | --- | --- | --- | --- |
| acarbose | 2.468 | -0.442 | 1.435 | linezolid | 0.220 | -3.712 | 0.151 |
| afatinib | 0.381 | -2.388 | 0.330 | lisinopril | 0.393 | -1.866 | 0.371 |
| alectinib | 2.390 | -0.481 | 1.398 | loratadine | 0.101 | -4.825 | 0.069 |
| ambrisentan | 0.039 | -5.486 | 0.035 | lorazepam | 0.164 | -4.132 | 0.112 |
| armodafinil | 1.109 | -1.469 | 0.716 | losartan | 0.817 | -1.001 | 0.741 |
| asenapine | 1.301 | -1.258 | 0.827 | macitentan | 0.154 | -3.299 | 0.144 |
| atomoxetine | 0.205 | -3.811 | 0.141 | minoxidil | 0.270 | -2.310 | 0.259 |
| azithromycin | 0.518 | -1.942 | 0.392 | naproxen | 0.868 | -0.849 | 0.795 |
| bicalutamide | 0.806 | -1.901 | 0.532 | ondansetron | 0.641 | -1.585 | 0.557 |
| bimatoprost | 0.187 | -3.944 | 0.128 | osimertinib | 0.159 | -3.875 | 0.128 |
| binimetinib | 0.493 | -2.579 | 0.333 | oxymetazoline | 1.043 | -1.551 | 0.677 |
| capecitabine | 0.122 | -3.709 | 0.114 | palbociclib | 1.910 | -0.762 | 1.158 |
| cefdinir | 1.781 | -0.850 | 1.091 | paroxetine | 0.322 | -2.869 | 0.257 |
| celecoxib | 0.577 | -1.288 | 0.545 | pimavanserin | 0.072 | -5.007 | 0.058 |
| citalopram | 0.299 | -2.402 | 0.278 | pirfenidone | 0.172 | -3.136 | 0.162 |
| clindamycin | 0.506 | -2.543 | 0.342 | pomalidomide | 0.066 | -4.650 | 0.061 |
| clonidine | 1.337 | -1.222 | 0.848 | raloxifene | 0.354 | -2.733 | 0.283 |
| crizotinib | 0.187 | -3.946 | 0.128 | ranitidine | 0.083 | -4.798 | 0.067 |
| dalfampridine | 0.119 | -3.579 | 0.114 | ranolazine | 0.142 | -4.339 | 0.097 |
| dapagliflozin | 0.271 | -3.111 | 0.218 | regorafenib | 0.218 | -3.728 | 0.149 |
| desvenlafaxine | 0.309 | -2.926 | 0.247 | riociguat | 0.171 | -3.764 | 0.138 |
| diazepam | 0.210 | -3.236 | 0.183 | roflumilast | 1.382 | -1.178 | 0.873 |
| dimethyl fumarate | 1.368 | -1.191 | 0.866 | ropinirole | 1.116 | -1.148 | 0.843 |
| docetaxel | 0.128 | -3.488 | 0.122 | rotigotine | 0.407 | -2.845 | 0.277 |
| doxycycline | 0.742 | -1.197 | 0.632 | rucaparib | 0.212 | -3.458 | 0.171 |
| entecavir | 0.565 | -1.932 | 0.470 | sildenafil | 0.335 | -3.121 | 0.228 |
| epirubicin | 0.609 | -2.284 | 0.409 | simeprevir | 0.679 | -1.503 | 0.589 |
| eplerenone | 1.306 | -1.253 | 0.830 | sitagliptin | 0.421 | -1.857 | 0.393 |
| erythromycin | 0.501 | -2.247 | 0.396 | sunitinib | 0.089 | -4.562 | 0.076 |
| escitalopram | 0.238 | -2.930 | 0.214 | tadalafil | 0.093 | -4.497 | 0.079 |
| febuxostat | 0.491 | -2.033 | 0.422 | temozolomide | 0.163 | -4.139 | 0.112 |
| fexofenadine | 0.128 | -4.177 | 0.103 | tetrabenazine | 0.330 | -3.141 | 0.225 |
| fluconazole | 2.484 | -0.110 | 1.703 | ticagrelor | 0.181 | -3.277 | 0.164 |
| fluoxetine | 0.245 | -2.802 | 0.223 | topiramate | 0.415 | -1.878 | 0.387 |
| fostamatinib | 0.826 | -1.868 | 0.545 | tramadol | 0.181 | -3.547 | 0.153 |
| gabapentin | 0.261 | -2.370 | 0.250 | triptorelin | 1.436 | -1.128 | 0.903 |
| glycopyrrolate | 0.666 | -1.704 | 0.550 | umeclidinium | 0.654 | -1.729 | 0.541 |
| indacaterol | 1.324 | -1.235 | 0.840 | ursodiol | 2.165 | -0.007 | 1.690 |
| ivacaftor | 0.345 | -3.077 | 0.236 | vismodegib | 0.292 | -2.863 | 0.247 |
| ixazomib | 0.233 | -3.632 | 0.160 | voriconazole | 0.227 | -3.672 | 0.155 |
| lapatinib | 0.390 | -2.905 | 0.266 | vortioxetine | 0.178 | -3.566 | 0.151 |
| latanoprost | 0.136 | -4.398 | 0.093 | warfarin | 0.117 | -4.165 | 0.100 |
| lenvatinib | 0.198 | -3.32 | 0.173 | zolpidem | 0.280 | -2.828 | 0.243 |
| levocetirizine | 0.317 | -3.198 | 0.216 |  |  |  |  |

*The calculated IC975 values for each drug at less than 0 are listed. RORUCI, the upper limit of the 95% confidence interval for the reporting odds ratio; IC975, the upper limit of the 95% confidence interval for the information component; EBGM95, the upper limit of the 90% confidence interval for the empirical Bayes geometric mean

Supplementary Table S3. Information about the gene expression datasets related to psoriasis from the GEO

| GSE ID | Platform | Number of Sample (psoriasis lesion/normal lesion) |
| --- | --- | --- |
| GSE136757 | Affymetrix Human Genome U133 Plus 2.0 Array | 30/30 |
| GSE117239 | Affymetrix Human Genome U133 Plus 2.0 Array | 83/84 |
| GSE117468 | Affymetrix Human Genome U133 Plus 2.0 Array | 128/128 |
| GSE85034 | Illumina Human HT-12 V4.0 expression beadchip | 30/30 |
| GSE78097 | Affymetrix Human Genome U133 Plus 2.0 Array | 27/6 |
| GSE79704 | Affymetrix Human Gene 2.1 ST Array | 12/20 |
| GSE121212 | Illumina HiSeq 2500 | 37/37 |
| GSE109248 | Illumina HumanHT-12 V4.0 expression beadchip | 17/14 |

GSE, Gene Expression Omnibus Series Experiment; GPL, Gene Expression Omnibus platform

Supplementary Table S4. Quality control results using

| GSE ID | IQC | EQC | CQCg | CQCp | AQCg | AQCp | Rank |
| --- | --- | --- | --- | --- | --- | --- | --- |
| GSE136757 | 6.51 | 3.52 | 307.65 | 307.65 | 7.05 | 206.62 | 1.58 |
| GSE117239 | 6.14 | 3.35 | 307.65 | 307.65 | 5.28 | 182.58 | 2.75 |
| GSE85034 | 4.44 | 3.40 | 307.65 | 307.65 | 6.92 | 168.27 | 2.83 |
| GSE117468 | 5.91 | 3.35 | 307.65 | 307.65 | 2.14* | 155.5 | 3.58 |
| GSE121212 | 3.63 | 2.19* | 273.66 | 274.02 | 3.04 | 151.13 | 5.33 |
| GSE78097 | 3.89 | 3.05 | 197.36 | 215.56 | 1.70* | 99.04 | 5.83 |
| GSE79704 | 1.11* | 3.52 | 52.52 | 101.09 | 0.18* | 72.57 | 6.25 |
| GSE109248 | 0.03* | 2.00* | 10.69 | 45.32 | 0.64* | 19.70 | 7.83 |

GSE, Gene Expression Omnibus Series Experiments; IQC, Internal quality control; EQC, External quality control; CQCg, Constituency quality control in genes; CQCp, Consistency quality control in pathway, AQCg, Accuracy quality control in genes; AQCp, Accuracy quality control in pathways; Rank, a standardized mean rank; **P* value not significant after a Bonferroni correction

Supplementary Table S5. Disease gene expression signatures for psoriasis

| GeneID | symbol | baseMean | | log 2 (fold change) | *P* value (×10^-20^) | Adjusted *P* value (×10^-20^) |
| --- | --- | --- | --- | --- | --- | --- |
| 416 | *ARSF* | 0.920 | | 2.654 | 1.00 | 1.53 |
| 479 | *ATP12A* | -2.680 | | 2.761 | 1.00 | 1.53 |
| 898 | *CCNE1* | 0.916 | | 2.876 | 1.00 | 1.53 |
| 1382 | *CRABP2* | 0.667 | | 3.011 | 1.00 | 1.53 |
| 1408 | *CRY2* | 1.581 | | -2.669 | 1.00 | 1.53 |
| 1824 | *DSC2* | -0.884 | | 2.800 | 1.00 | 1.53 |
| 1830 | *DSG3* | 0.100 | | 2.573 | 1.00 | 1.53 |
| 1992 | *SERPINB1* | -0.676 | | 2.631 | 1.00 | 1.53 |
| 2171 | *FABP5* | -1.111 | | 3.076 | 1.00 | 1.53 |
| 2524 | *FUT2* | 0.218 | | 2.637 | 1.00 | 1.53 |
| 3838 | *KPNA2* | 0.821 | | 2.990 | 1.00 | 1.53 |
| 3853 | *KRT6A* | -1.034 | | 2.573 | 1.00 | 1.53 |
| 3854 | *KRT6B* | 0.408 | | 2.523 | 1.00 | 1.53 |
| 3868 | *KRT16* | -0.424 | | 3.126 | 1.00 | 1.53 |
| 3934 | *LCN2* | 2.699 | | 2.618 | 1.00 | 1.53 |
| 4067 | *LYN* | -1.462 | | 2.542 | 1.00 | 1.53 |
| 4084 | *MXD1* | 0.491 | | 2.515 | 1.00 | 1.53 |
| 4599 | *MX1* | 0.902 | | 2.693 | 1.00 | 1.53 |
| 4644 | *MYO5A* | -0.133 | | 2.521 | 1.00 | 1.53 |
| 4860 | *PNP* | 0.521 | | 2.821 | 1.00 | 1.53 |
| 4938 | *OAS1* | 1.310 | | 2.507 | 1.00 | 1.53 |
| 4939 | *OAS2* | 0.027 | | 2.715 | 1.00 | 1.53 |
| 4940 | *OAS3* | 1.028 | | 2.724 | 1.00 | 1.53 |
| 5266 | *PI3* | -0.045 | | 2.860 | 1.00 | 1.53 |
| 5275 | *SERPINB13* | 0.881 | | 3.414 | 1.00 | 1.53 |
| 5873 | *RAB27A* | 3.587 | | 2.641 | 1.00 | 1.53 |
| 6241 | *RRM2* | 0.794 | | 2.707 | 1.00 | 1.53 |
| 6280 | *S100A9* | 3.286 | | 3.018 | 1.00 | 1.53 |
| 6317 | *SERPINB3* | -0.182 | | 3.420 | 1.00 | 1.53 |
| 6318 | *SERPINB4* | -1.868 | | 3.695 | 1.00 | 1.53 |
| 6641 | *SNTB1* | 1.617 | | -2.607 | 1.00 | 1.53 |
| 6648 | *SOD2* | 0.768 | | 2.733 | 1.00 | 1.53 |
| 7051 | *TGM1* | 0.188 | | 2.958 | 1.00 | 1.53 |
| 7326 | *UBE2G1* | 0.564 | | 2.514 | 1.00 | 1.53 |
| 7378 | *UPP1* | -0.398 | | 2.884 | 1.00 | 1.53 |
| 7466 | *WFS1* | 1.237 | | -2.595 | 1.00 | 1.53 |
| 7474 | *WNT5A* | -0.034 | | 3.096 | 1.00 | 1.53 |
| 8111 | *GPR68* | 0.136 | | 2.695 | 1.00 | 1.53 |
| 8851 | *CDK5R1* | -0.617 | | 2.948 | 1.00 | 1.53 |
| 9517 | *SPTLC2* | -0.253 | | 2.987 | 1.00 | 1.53 |
| 9635 | *CLCA2* | 0.815 | | 2.782 | 1.00 | 1.53 |
| 10135 | *NAMPT* | 2.321 | | 2.606 | 1.00 | 1.53 |
| 10200 | *MPHOSPH6* | -1.884 | | 2.516 | 1.00 | 1.53 |
| 10205 | *MPZL2* | 0.471 | | 2.966 | 1.00 | 1.53 |
| 10622 | *POLR3G* | -1.180 | | 3.104 | 1.00 | 1.53 |
| *(continued)* | | | | | | |
| 10804 | *GJB6* | -0.011 | | 3.027 | 1.00 | 1.53 |
| 10855 | *HPSE* | -1.161 | | 2.757 | 1.00 | 1.53 |
| 10890 | *RAB10* | 1.532 | | 2.504 | 1.00 | 1.53 |
| 23242 | *COBL* | 1.359 | | -2.636 | 1.00 | 1.53 |
| 23589 | *CARHSP1* | 1.425 | | 2.920 | 1.00 | 1.53 |
| 26064 | *RAI14* | 0.960 | | -2.590 | 1.00 | 1.53 |
| 51458 | *RHCG* | -1.152 | | 3.104 | 1.00 | 1.53 |
| 55008 | *HERC6* | 1.310 | | 2.551 | 1.00 | 1.53 |
| 55165 | *CEP55* | | 0.087 | 2.512 | 1.00 | 1.53 |
| 55803 | *ADAP2* | | -0.596 | 2.750 | 1.00 | 1.53 |
| 56300 | *IL36G* | | -1.719 | 3.253 | 1.00 | 1.53 |
| 56967 | *C14ORF132* | | 1.904 | -2.543 | 1.00 | 1.53 |
| 57016 | *AKR1B10* | | 1.795 | 3.191 | 1.00 | 1.53 |
| 64785 | *GINS3* | | 0.640 | 3.010 | 1.00 | 1.53 |
| 79153 | *GDPD3* | | 2.677 | 2.636 | 1.00 | 1.53 |
| 79887 | *PLBD1* | | 1.201 | 2.934 | 1.00 | 1.53 |
| 81671 | *VMP1* | | 1.557 | 2.531 | 1.00 | 1.53 |
| 84632 | *AFAP1L2* | | -0.611 | 2.845 | 1.00 | 1.53 |
| 84817 | *TXNDC17* | | 2.188 | 2.556 | 1.00 | 1.53 |
| 89894 | *TMEM116* | | -0.262 | -2.752 | 1.00 | 1.53 |
| 115019 | *SLC26A9* | | 1.789 | 2.782 | 1.00 | 1.53 |
| 131583 | *FAM43A* | | 0.707 | 2.971 | 1.00 | 1.53 |
| 140739 | *UBE2F* | | -1.916 | 2.815 | 1.00 | 1.53 |
| 171546 | *SPTSSA* | | 1.719 | 2.534 | 1.00 | 1.53 |
| 283748 | *PLA2G4D* | | 0.306 | 3.719 | 1.00 | 1.53 |
| 374454 | *KRT77* | | -1.203 | -2.880 | 1.00 | 1.53 |
| 387695 | *C10ORF99* | | -0.194 | 2.629 | 1.00 | 1.53 |
| 642273 | *FAM110C* | | 0.664 | 2.717 | 1.00 | 1.53 |

Log_2_ (fold change)>2.5 and adjusted *P* value<0.001 were used to identify significantly differentially expressed genes between psoriatic and normal lesions.

## Supplementary Figures

**Supplementary Figure S1.** Principle component analysis of the quality control set, including IQC, EQC, AQCg, AQCp, CQCg, and CQCp measures in the eight disease from the Gene Expression Omnibus (GEO) Series Experiments (GSEs) by MetaQC. IQC, internal quality control; EQC, external quality control; AQCg, accuracy quality control of the featured genes; AQCp, accuracy quality control of the pathway; CQCg, consistency quality control in the ranking of the featured genes; CQCp, consistency quality control in the ranking of the pathway. 1, GSE136757; 2, GSE117239; 3, GSE85034; 4, GSE117468; 5, GSE121212; 6, GSE78097; 7, GSE79704; 8, GSE109248.


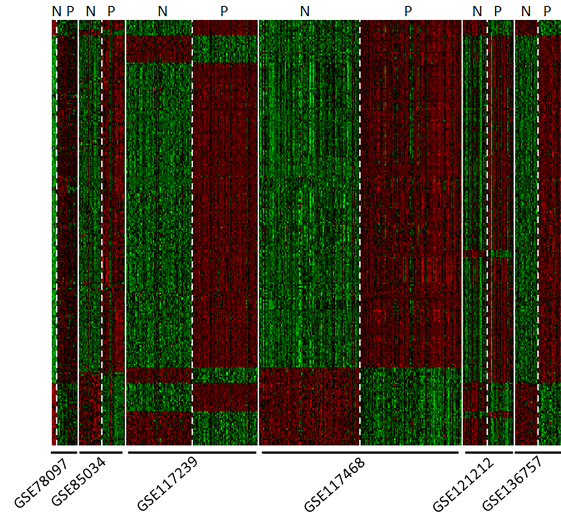


**Supplementary Figure S2.** Heatmap of expression profiles of the top up- and downregulated differentially expressed genes from the meta-analysis of psoriasis. Genes with log2fold changes exceeding 2.0 with the false discovery rate set to <0.001: upregulated genes shown in red; downregulated genes shown in green. N, normal; P, psoriasis, GSE, Gene Expression Omnibus Series Experiments.
